# Perceptions and experiences of environmental health and risks among Latina mothers in urban Los Angeles, California, USA

**DOI:** 10.1186/s12940-023-00963-2

**Published:** 2023-01-14

**Authors:** Elizabeth M. Kamai, Andrea Calderon, Yoshira Ornelas Van Horne, Theresa M. Bastain, Carrie V. Breton, Jill E. Johnston

**Affiliations:** 1 Department of Population and Public Health Sciences, Keck School of Medicine, University of Southern California, Los Angeles, CA USA; 2 Department of Environmental Health Sciences, Mailman School of Public Health, Columbia University, New York, NY USA

**Keywords:** Perceptions, Environmental health, Latina, Mothers, Pregnancy

## Abstract

**Background:**

Environmental exposures during pregnancy and early childhood can have acute and chronic adverse health impacts. As minoritized populations are more likely to reside in areas with greater pollution, it is important to understand their views and lived experiences to inform action. The purpose of this community-driven qualitative research study was to understand how urban Latina mothers in Los Angeles County, California perceived environmental health and risks.

**Methods:**

We conducted semi-structured individual interviews with Latina pregnant women and mothers of young children, recruited through existing collaborations with community organizations. Interviews conducted in either English or Spanish and were coded inductively according to a modified grounded theory approach.

**Results:**

Thirty-six Latina mothers completed interviews between August–October 2016. Participants lived primarily in low-income communities of South-Central Los Angeles and East Los Angeles. We identified three major themes based on the participants’ responses during interviews: *Defining the Environment*, *Environment & Health Risks*, and *Social & Political Responsibility*. Women defined their environment in terms of both “nature” and “hazards.” They consistently identified foul odors, dirtiness, noise, trash, bugs, smoke, and other visible blights as indicators of household and neighborhood environmental hazards. They expressed fear and uncertainty about how their environment could affect their health and that of their children, as well as specific concerns about respiratory health, asthma, allergies, cancer, and adverse pregnancy outcomes. Mothers often changed individual behaviors around diet and cleaning during pregnancy but were frustrated by power imbalances that left them unable to change their home or neighborhood environments, despite their desire to do so.

**Discussion:**

Our study is among the first to describe how urban Latina mothers perceive and experience environmental health risks during pregnancy and early childhood. Our research suggests additional attention is needed by public health professionals and researchers to address the environmental health risks that matter most to urban Latina mothers. They also highlight the tension that many urban Latina mothers feel between wanting to protect their families’ health and well-being and feeling powerless to change their environment. Broad policy changes, rather than additional individual recommendations, are needed to address the concerns of this vulnerable population.

**Supplementary Information:**

The online version contains supplementary material available at 10.1186/s12940-023-00963-2.

## Background

Environmental insults that occur during pregnancy or early childhood can adversely affect a range of reproductive, pregnancy, and childhood health outcomes [1–4]. Immigrant, low income, and women of color are doubly vulnerable during pregnancy as they frequently bear a disproportionate burden of exposure to toxic chemicals [5, 6].

Given the vulnerability of infants to environmental contaminants [7], the disproportionate role that mothers play in managing household activities and family health [8], and the relative frequency with which pregnant people interact with health professionals, pregnant people and new mothers are often the key audience for media and public health campaigns. Further, pregnancy is seen as a key time for interventions designed to reduce environmental health disparities [9].

Understanding how pregnant people and new parents perceive and respond to risks is critical for developing effective risk communication strategies, yet limited research has been done in this context [8]. We know little about how individuals, particularly new mothers, may perceive and experience the numerous contaminant exposures in their everyday lives, especially in communities facing cumulative burdens of many sources of environmental exposures.

There are unique concerns specific to low-income people of color and immigrant communities, who disproportionately face multiple and complex environmental and social exposures due to structural and environmental racism, coupled with fear or mistrust of government. Inner cities across America are often sites of concentrated poverty and dumping grounds for locally unwanted land uses and industries [10]. In many cases, these fenceline industries are un- or under-regulated [10]. These hazards are amplified by other negative socioeconomic and health factors, including higher rates of chronic diseases, lack of access to healthy foods, substandard housing, and stress from racism, poverty, unemployment, and crime [11]. Racist policies such as housing segregation and disproportionate siting of toxic facilities in low income, Black and Latinx neighborhoods are important factors in understanding who is impacted by environmental health problems such as poor air quality (especially for children), incompatible land use (including the lack of regulations resulting in schools in low-income neighborhoods being built near freeways, or next to toxic industries), or inadequate green space in urban neighborhoods [10, 12–15].

In Los Angeles County, as elsewhere in California, Latinos often reside in environmental justice communities, that is, neighborhoods disproportionately burdened by environmental risks and pollution, with fewer environmental amenities, and more vulnerable to environmental hazards [5, 12, 13, 16–20]. This higher cumulative environmental risk may contribute to observed health disparities among Latino children, particularly higher rates of obesity, diabetes, and asthma [21–24]. In collaboration with community-based organizations, we designed this community-driven qualitative research study to understand environmental health and risk perceptions among new urban Latina mothers using semi-structured interviews. Interviews provide contextually rich descriptive data, foster interactive research, and offer greater possibilities for participants to share their life experiences, attitudes, values, and perceptions. Qualitative research can provide unique in-depth insights on such dynamic interactions and offer community-level perceptions of environmental disamenities and their relationship to behavior [25–27].

Our primary objectives were to: investigate new mothers’ experiences, perceptions, and meanings of environmental health risks to their children; explore behavioral responses to perceived risks; and examine potential barriers and facilitators to taking protective actions to minimize risks.

## Methods

We conducted a series of semi-structured individual interviews with Latina pregnant women and mothers of young children in collaboration with local community organizations. We recruited volunteer participants using existing collaborations with community organizations in South and East Los Angeles neighborhoods through neighborhood meetings and community events.

Mothers of young children (and mothers-to-be) were asked to participate in a ~ 1 hour interview during the period from August to October 2016. Participants were required to identify as Hispanic/Latina, speak English or Spanish, be over 18 years old, and either be pregnant or have a child 5 years of age or younger. Interviews were conducted in English or Spanish at home or in a central agreed-upon private location (like a meeting room at a community center or library).

We created a discussion guide (Additional file 1) for semi-structured interviews to follow that included: perceived environmental risks specific to home, neighborhood, and workplace; the role of environmental hazards on health; preventative actions taken and considered; facilitators and barriers to actions; sources of information on environment and environmental health; use and interest in scientific research and studies. To avoid alarming or leading participants, no questions about any *specific* environmental or health issues were asked, but specific issues were discussed if raised by the participant themselves. The study was approved by the Institutional Review Board at the University of Southern California.

Interviews were conducted in both English and Spanish by two bilingual women. They were digitally recorded, and transcripts were entered into a qualitative data analysis software (Atlas.ti, Scientific Software Development, Berlin) for coding and analysis purposes. Interviews were transcribed and coded in their original language by two staff members who identified as bilingual and bicultural Latina women. These staff members and an interviewer together developed a detailed coding scheme with top codes and sub codes in an iterative, modified grounded theory approach [28, 29]. The two coders began with a list of deductive codes based on the interview guide. After an initial review of the interviews, they inductively developed additional codes with an interviewer and iterated those codes as they analyzed the data, identifying areas of overlap and divergence among participants [30]. We conducted a reliability check on a sample of five interviews by calculating the intracoder agreement (ICA) as the number of times a set of ratings are the same, divided by the total number of units of observation that are rated, multiplied by 100. The two coders agreed approximately 78% of the time, with discrepancies largely based on the length of the text quoted. In areas where the coders did not agree, a consensus review process was completed to address any discrepancies in consultation with one of the interviewers.

## Results

**Fig. 1:**
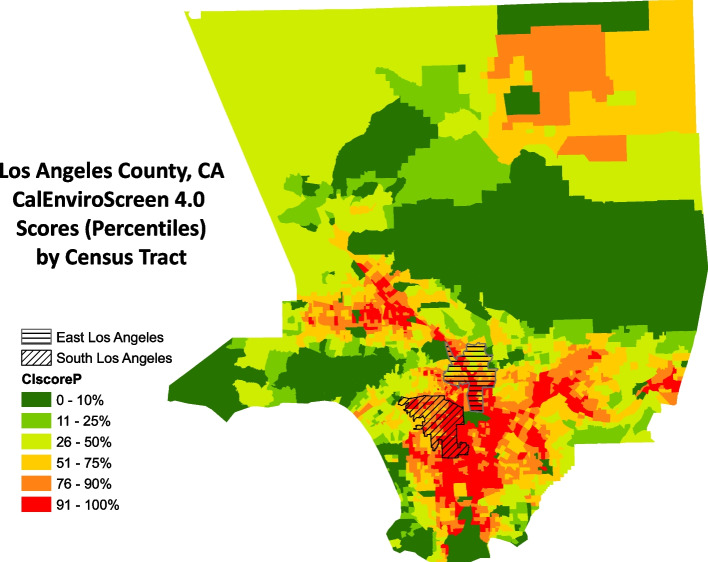
Map of CalEnviroScreen 4.0 percentiles (higher percentile indicates poorer cumulative environmental burden) of Census tracts in Los Angeles County, with the South Los Angeles and East Los Angeles planning areas shaded

We conducted a total of 36 semi-structured interviews among Latina mothers living in urban Los Angeles County, California from August–October 2016. All participants identified as Latina women and mothers and were the birth parent of their child (ren). Participants lived primarily in South-Central Los Angeles or East Los Angeles – low-income communities, disproportionately burdened by environmental pollution, as indicated by the CalEnviroScreen, version 4.0 [31] (Fig. 1). Most of the interviews were conducted in Spanish (27/36), and the majority of participants were born outside of the United States (23/36) (Table 1). The median age of participants was 32.5 years. All had between one and five children, with a median age of 2.5 years for the youngest child, and four women reported being pregnant at the time of their interview. Less than a third of women (10/36) reported occupations outside of the home, which included housecleaning, garment work, and fast-food restaurants.

**Table 1:** Demographic characteristics of study participants

Characteristic	*n* (%)
Total	36 (100%)
Age	
18–24	7 (19%)
25–35	18 (50%)
36–45	11 (31%)
Currently pregnant	4 (11%)
Number of children	
1	9 (25%)
2	10 (28%)
3 or more	16 (44%)
Country of origin	
U.S.	13 (36%)
Mexico	17 (47%)
Other non-U.S.	6 (17%)
Language of interview	
Spanish	25 (69%)
English	11 (31%)

**Fig. 2:**
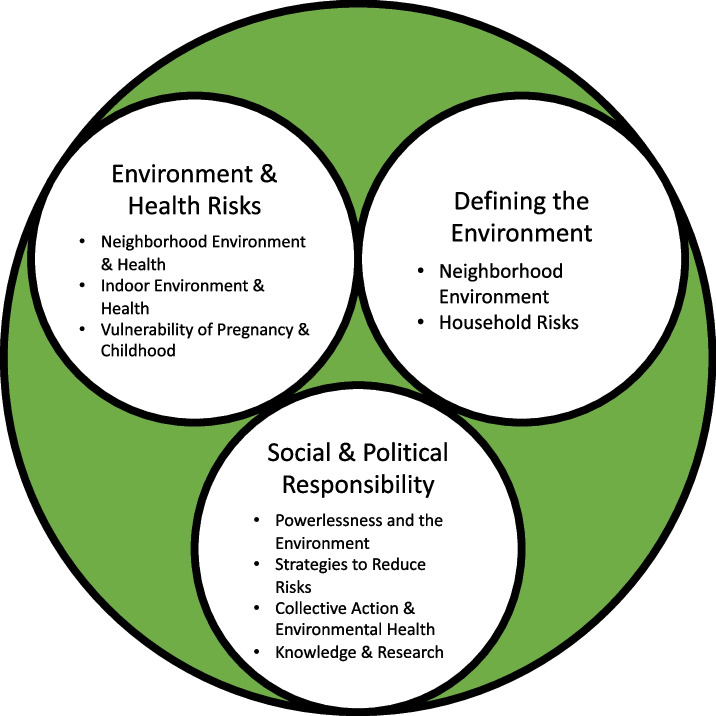
Themes and Subthemes identified in interviews

We identified three major themes based on the participants’ responses during interviews (Fig. 2): *Defining the Environment*, *Environment & Health Risks*, and *Social & Political Responsibility*. While quotes from interviews conducted in Spanish were translated for this article, the original quotes are available in Additional file 2.

### Defining the environment

Participants were asked about their perceptions of the environment, environmental health, and sources of environmental pollution, toxins, or hazards. The concept of environment and environmental health raised diverse ideas across participants. The majority offered a definition of their environment, frequently describing the environment as a natural or green space. There was a notable divide between women who defined environment in terms of neighborhood amenities, cleanliness, or safety, and those who used terms relating to hazards, dirtiness, or disamenities. For a few, the environment was a source of health or safety:

*Well, for example that the community is clean. That it be safe for families and the kids.* (age 23, translated from Spanish).

However, many other participants described their environment as a source of physical or chemical hazards:

*What I sometimes think about is cigarettes, and drugs, and gangs, and bullets. Sometimes about the water, it sometimes arrives to us dirty.* (age 28, translated from Spanish).

Three women referenced the climate crisis in association with their perception of the environment. Women who had recently immigrated from outside the United States sometimes described the environment in their Los Angeles neighborhood in comparison to that in their country of origin, describing Los Angeles as having the worse environment.

Based on participants’ responses, we further created two subthemes within the theme of Defining the Environment: *Neighborhood Environment* and *Household Risks*.

#### Neighborhood environment

When asked about environmental concerns in their neighborhoods, many participants described tangible or visible perceived blights or nuisances. Two thirds of mothers interviewed expressed that the presence of trash near their homes or community was an environmental concern.

*I think just sanitation wise …there was always trash on the floor, you can’t just sit on a sidewalk and feel comfortable. It would be disgusting… You can’t even sit on a bus bench because it is so gross. So I think you know walking out, aside from safety, it’s just, it’s not a pretty sight.* (age 22).

Most participants perceived contaminated air as an environmental hazard in their neighborhoods. Concern about “smog” was common and used to describe poor air quality, traffic pollution or vehicle exhaust. A 40 year-old woman described the “bad air quality” around “houses that are very close to the freeway that are smelling all that smog and the pollution from the cars.” Some women also provided specific examples of stationary sources, particularly factories and industrial sites, that contributed to air pollution in their environments.

Many mothers were concerned by the presence of undesirable odors, especially those over which they had no ability to mitigate or stop. For one 31-year-old mother, smells were a key distinguishing feature between the “healthy” environment of “the forest” and that of her neighborhood. Women frequently described odors from cigarettes and marijuana in public spaces, such as bus stations, as well as odors from potentially harmful industries in their neighborhoods.

*When you go out on the street you always see people smoking at the bus stops, and everywhere you go outside you always see people smoking. And even though one may want to avoid it, one always finds people smoking. I think this is the problem.* (age 32, translated from Spanish).

Two participants also described how noise from traffic and construction contributed to their environments feeling less safe and healthy. Experiences of bad odors sometimes overlapped with concerns about smog and visible smoke.

#### Household risks

Participants perceived indoor spaces as important potential sources of environmental health risks for themselves and their families. Many women linked lack of visible contamination or pests in their homes to a less hazardous indoor environment, and inversely, described features of their households that contributed to dirtiness, and what they perceived as therefore poorer environmental quality. They were frustrated by structural barriers to what they perceived as an environmentally healthy home interior.

The presence of lead in participants’ homes was a common concern, frequently linked to more general concerns about cleanliness and upkeep of their homes or apartments. A 45 year old mother of three described how her concerns about peeling paint and dust on a dirty carpet were related to her children’s health:

*The paint, well, pieces are falling off and the manager does not want to repaint. And I see my child sometimes peel the paint...Regarding my health, yes [I am worried], because of the dust...sometimes something falls and the children put it in their mouths without us noticing...This is dangerous, and that is why there is worry*. (age 45, translated from Spanish).

Similarly, four women expressed concern about mold in their living spaces, usually in the context of lack of cleanliness or blights on their walls. Several participants also described how various kinds of pests – including cockroaches, mosquitoes, and bedbugs – and rodents were environmental hazards in their homes. On the other hand, women voiced concerns about pesticides causing health problems. Two described how the chemicals used to control pests in and around their living spaces were toxic to children:

*The pesticides – the ones that kill insects? It causes harm to the brain... that is why it kills animals fast and is dangerous for children.* (age 34, translated from Spanish).

In tension with concerns about cleanliness was the nearly universal awareness of the hazards of chemicals in common household cleaning products. Women described how strong-smelling cleaning products were the most harmful, again highlighting the perceived link between odor and hazards. When asked about household concerns, one 19 year old mother said:

*Well, I think for example, most people the way that they are most exposed to chemicals is cuz of the cleaning supplies that they use. So like changing the type of cleaning supplies would be one thing because there is some that are better for you and don’t have that really potent smell.* (age 19).

About half of participants were concerned about the quality of their drinking water at home. Again, women were attuned to tangible experiences with odor, color, and cleanliness when describing their concerns with water.

*[The tap water] is disgusting…I see the warm water come in and I see kinda like different colors come out…like yellow*. (age 30).

Two different participants described becoming ill as a result of using the tap water in their home, with one woman directly linking “not clean” to “harm”:

*The water also harms us. If it [the water] is not clean, like when we shower or drink it, it can harm our body system like causing vomiting, diarrhea, and fever.* (age 31, translated from Spanish).

### Environment & Health Risks

The second overarching theme we identified throughout the interviews was that of Environment & Health Risks. Participants were particularly vocal about how their outdoor environment was linked to various health concerns during pregnancy, in childhood, and in adulthood.

#### Neighborhood Environment & Health

Outdoor air quality dominated women’s descriptions of how their environments could affect their health; participants perceived a range of health harms due to exposure to smog, smoke, and bad odors. Some participants described potential health concerns in general terms:

*If there is a lot of smog, and all that, well it is not healthy.* (age 34, translated from Spanish).

Many participants described how exposure to smog, smoke, and odors were related specifically to respiratory health problems such as asthma and allergies. Two participants were concerned that air pollution or poor air quality could result in cancer in their communities.

*I think that all that smoke can harm a person. Then suddenly it affects one’s health because it triggers asthma and a lot of allergies.* (age 35, translated from Spanish).

Women sometimes described how the smell of trash could affect their health. One mother described how the “aroma” coming from the garbage cans could be “toxic”:

*[B]ecause there is a lot of viruses from bugs, mosquitos that are around... and then they bring allergies.* (age 35, translated from Spanish).

Some mothers linked the presence of trash in their neighborhood to the presence of “la gente homeless” or “los homeless” – unhoused people living on streets. A 34 year old mother felt that these populations left trash on the street, which in turn caused health problems for both her family and the community at large.

Finally, one woman described the mental health impacts of environmental hazards.

*My experience is that when the environment is not in good conditions sometimes it affects our personality, it affects the way we feel. I relate to that, when I see a bad habit we get sick or get depressed because of the environmental changes*. (age 21).

#### Vulnerability of Pregnancy & Childhood

All participants in this study were either pregnant or had been pregnant in the past five years, and many perceived pregnancy as a particularly vulnerable period for exposure to environmental toxins.

*[P]regnancy is very delicate. One has to be very careful when pregnant to protect the baby and oneself.* (age 40, translated from Spanish).

Participants described a variety of potential reproductive issues related to pollution and environmental hazards, including miscarriages or premature delivery. Several women described how maternal health and adverse exposures during pregnancy were linked to the health of the fetus and child.

*It affects them in many aspects. For example, there are women who can’t breathe when they smell chemicals. And by not breathing, it affects the baby. Because the moment that you are breathing too hard the baby will be agitated inside.* (age 34, translated from Spanish).

Some women expressed concerns about how exposure to environmental hazards during pregnancy – particularly smoke, smog, and chemicals with strong odors like bleach and gas from stoves – could cause birth defects, “deformities or malformations,” and problems related to brain development.

*I think that [environmental hazards] can affect babies very much. They can be born with a malformation or it can hinder their growth...it can affect them very much*. (age 36, translated from Spanish).

Three mothers also described concerns about how chemicals in consumer products like plastics could cause cancer in their children or families.

Others were concerned about environmental hazards during pregnancy but expressed uncertainty about specific health effects. Lack of information contributed to their concerns. One woman described how she was worried about contamination in soil affecting pregnant women:

*I feel that somehow whatever is around us is being affected. If it affects the soil it’s gonna affect the mothers somehow… You know, that the thing, that I don’t know. I don’t know, I feel it will affect her somehow. Maybe neurologically… Depending on how, also, how long the mother has lived there. And how long has this been happening for...I just don’t know how.* (age 40).

There were also participants who, despite describing environmental concerns in their home or neighborhood, stated they personally had no such health concerns during pregnancy, such as a 35-year-old mother who responded:

*Well no. Everything is normal. I didn’t feel it affect me*. (age 35, translated from Spanish).

### Social & Political Responsibility

The final theme we identified based on the interviews was Social & Political Responsibility. Within this theme, we created four subthemes: *Powerlessness and the Environment*, *Strategies to Reduce Risk*, *Collective Action and Environmental Health*, and *Knowledge & Research*.

#### Powerlessness and the environment

Women frequently described situations in which they felt they were powerless to control their environment and faced obstacles to avoiding environmental health hazards.

Several were frustrated by smoke and odors in their broader neighborhood environments that came from a variety of sources. A 36 year old mother of three described how smokers and traffic pollution at the bus stop caused her stress and were an exposure she could not avoid:

*Secondhand smoke affects those who do not smoke more than those who smoke. People know that but do not respect it. They do not respect that they are causing harm to other people. The other [source of pollution] is contaminants from cars but you can’t avoid that. This city is very contaminated by cars.* (translated from Spanish).

A 31 year old mother of two described how all the smell from tar, smoking, and marijuana gets in her house, and all you can do is “close the windows” or “cover yourself when we’re on the street,” but that “[w]e can’t avoid [the smog].”

Some women felt they lacked agency in their own homes because power was held by landlords or management companies, who displayed negligence when they communicated their concerns about their living conditions. A 40 year old mother of three described the lengths she went to in order to prevent her home from being fumigated:

*They tried to enter my apartment to fumigate, but I didn’t let them because my doctor told me that it is bad for my kids and I. And they even sued me. They wanted to kick me out because I didn’t let them inside. But thank God, I was protected by the note my doctor gave me. And the lawsuit didn’t continue.* (age 40, translated from Spanish).

Several women also felt like their landlords did not care about their living conditions. A 36 year old mother described her frustration with how long it took for the building management to deal with holes in her walls through which mice were entering her apartment. Another participant shared that after an infestation of cockroaches and bed bugs, the owners took no action:

*[H]ere the managers of the apartments are...[laughing]...they don’t care...they only care about receiving rent.* (age 36, translated from Spanish).

Financial concerns or lack of resources contributed to several women’s feelings of powerlessness around environmental health risks. One 45 year old mother of three described how she tried to eat healthy food and go to the farmers market, but even with subsidies and coupons, it was too expensive:

*For all the deals they are having, the money does not go far enough. Because only two baskets of strawberries, or three, it is already five dollars. A spinach bunch is already two dollars. So, then the deal is useless because the money does not last.* (age 45, translated from Spanish).

Employment was another situation where women felt they were unable to prevent chemical exposures:

*I was working in garment factory and in that job the fabrics make a lot of dust so when you sew clothes it creates dust and sometimes the dust enters you through your nose, ears and eyes. And sometimes you find it hard to breathe. …No, they don’t give you anything [protective gear] and one earns very little. Because sometimes you earn five cents, ten cents, fifteen cents per piece and you do not make many pieces but you get full of dust.* (age 32, translated from Spanish).

There was repeated tension between health and resources on families, as described by a 29 year old mother of two:

*If they are always getting sick, the money. If you have the resources of course you are going to go to the doctors, but if you don’t, what do you do? It does affect, am I going to waste the food today or am I going to go to the doctor? Do I have enough to buy the medicine?* (age 29).

#### Strategies to reduce risks

Perhaps due to the various obstacles to controlling environmental health risks outside of their home, women concentrated their efforts on reducing perceived environmental health risks in their indoor home environments and on individual level changes they could make.

About a third of participants reported using filters with their tap water or purchased bottles or jugs of water rather than drinking tap water in their homes.

Two thirds of mothers described altering their behaviors around cleaning and cleaning product use, especially during and after pregnancy, to reduce their and their children’s exposure to chemicals in cleaning products. A few had family members clean their homes or bathrooms while they were pregnant, usually their husband or mother-in-law. One woman said she wore a mask when she used strong smelling cleaning products. Another reported cleaning only when her kids were in school, as well as keeping the baby in a separate room when she cleaned the bathroom. Others made efforts to keep bleach or other products out of reach of their children. One participant who used cleaning products at her place of work received a doctor’s note to limit her use of harmful chemicals at work. Several mothers described how they started using more natural cleaning products.

*Before I would buy more bleach for everything. Now I only use soap and baking soda.* (age 34, translated from Spanish).

However, there were a few women who did not change their cleaning product usage. A 40 year old mother of four was not convinced that alternative cleaning products would be effective.

*I mean I would try [natural homemade solutions], but I don’t know if they would work or not.* (age 40).

Others were concerned that natural or organic products were too expensive. A 32 year old mother of three explained that she knew she could reduce her chemical exposure by using different cleaning products:

*[B]ut the prices are very high and a low-income family cannot buy those products.* (age 32, translated from Spanish).

Other mothers reported cleaning more once their children were born. One 34 year old mother of four described specifically cleaning more frequently to prevent her children being exposed to lead. Similarly, a 30 year old mother described how she washes her hands more than she did before she was pregnant.

*Now, I wash my hands a lot. Especially [my son’s] hands because his little nails get dirty really fast.* (age 30).

Women also frequently described changing their diets during pregnancy by purchasing or eating “better” food for themselves or their families. Usually this involved buying organic products and eating more fruits and vegetables. A 34 year old mother said that during pregnancy she ate, “more organic, more fresh”:

*Especially if you’re pregnant, you have to be more careful what you’re eating. You know you can’t eat junk food. It’s better for pregnant women to eat fruit and vegetables… Whatever you eat, the baby eats. And you want the best for your baby.* (age 34).

Other women described limiting their intake of caffeine or soda, while one 30 year old mother of two said she was not eating chili because she was breastfeeding.

Some women started or stopped using other types of consumer products during pregnancy. Five English-speaking mothers described avoiding baby powder or talcum powder either during pregnancy or with their newborns out of concern that it caused cancer or breathing problems. A 37 year old mother described buying BPA-free plastics for heating food in the microwave. A 29 year old mother started using bug repellant because of concerns about Zika, while on the other hand, a 40 year old mother of five replaced pesticides with boric acid because it was “less toxic.”

#### Collective Action & Environmental Health

Few women specifically described how governmental entities or structures contributed to their neighborhood environmental health. For some, local authorities improved their neighborhood environment. One 30 year old mother of two described how the city of Los Angeles provided street sweeping and cleaned graffiti in her neighborhood. A 37 year old mother of two similarly felt that when there were potential environmental hazards in Los Angeles, the government tried to address them, whereas in her home country in Central America, companies were not held responsible for the pollution they caused. On the other hand, one woman felt that compared to her hometown, authorities in Los Angeles did not actively improve the environment:

*For example, I am from a town, where when they cut down a tree they plant 10 more trees. That way the ecosystem, the scenery keeps growing. Here when there are no trees there is nothing, they don’t do anything to change that.* (age 31, translated from Spanish).

One mother shared broader policy and environmental improvements she hoped to see:

*I would like them to remove the petroleum wells so that that there are no more contaminants… And the exhaust from the cars…All of this is polluting.* (age 36, translated from Spanish).

More frequently, though, women perceived environmental health as the result of local community or individual actions. They described ways that mothers, families, or neighborhoods could act to address environmental health concerns and improve their environment. Two women mentioned hybrid and electric cars, one mentioned carpooling, and a few suggested using bicycles as ways that individuals could address climate change or pollution from cars.

*If we all do something simple like not waste water, recycle. Run or use bicycles… The world would be better.* (age 21).

Water conservation was of particular concern to a few participants. Two young mothers described making changes to the way they wash dishes and bathe themselves and their children in order to conserve water.

*[T]here are places where a lot of water is wasted. They leave the water running all night…there is not much water and the water that can be consumed is running out.* (age 26, translated from Spanish).

A few participants spoke about hope or concerns regarding “global warming,” climate change, or “the future,” especially in reference to their children.

*I think I have to try not to throw things [away] for the good of the children because we already got to live but they still need a chance to live in a clean and healthy environment too.* (age 41. translated from Spanish).

A 30 year old mother explained:

*We need to take more pride in the place that we live and take care of it for the future.*

#### Knowledge & Research

Nearly all participants expressed an interest in learning more about improving environmental health, linking knowledge to opportunities for collective action and hope for the future. More than half of the women interviewed said they participated in this research study in order to learn more about the environment and connection to health. A few women went so far as to express disappointment that the interview process was not more educational.

*I would want to learn things that people can do to actually better the environment. Instead of just being told ‘oh, people hurt the environment’ be like, ok what can people do to make it better.* (age 19).

Several women said they chose to participate in the interviews because they thought it would help others, both directly by sharing information with other women, or indirectly.

*[B]ecause I imagine that the answers that one provides will be used to help the community with more programs.* (age 25, translated from Spanish).

Participants shared a variety of sources of knowledge, with little consistency about where women found environmental health information. Some women primarily relied on doctors or clinicians when they had questions or concerns about their children’s or their own health.

*It could be internet, it could be friends, it could be doctors. Or if I have a couple of doctor friends, I could ask them. Just send them a message…or TV shows like Dr. Oz.* (age 40).

Several women described learning about health, pregnancy, nutrition, and the environment through workshops and classes in parks, schools, or community centers. Several mothers described how friends or family members, particularly maternal figures (mothers, grandmothers, mothers-in-law), had shared their knowledge. Women also learned about health information from television and the internet. For some, the internet was more accessible than other news sources. One 27 year old mother of three described how she had learned about natural remedies on the internet, which changed how she interacted with the medical system:

*For example, to not go to the doctor. I think and I feel that medicine has a lot of chemicals. So I say no. Instead, I make a natural tea.* (age 27, translated from Spanish).

A few women, mostly younger moms born in the U.S., specifically described their attempts to find “accurate” or “reliable” information. An 18 year old new mom said she primarily relied on her doctor and health classes, rather than the “not totally accurate” news. Similarly, a 22 year old mother described how she used the information she found online, giving more credibility to websites from universities or nonprofits.

No participant in this study had previously participated in a research study, and women suggested a variety of reasons why others might be unwilling to do so. A key concern was the lack of free time for busy moms.

*But the other mothers, in contrast, are working. They focus only on work, then go home, and go to work, and home again. So that’s one of the factors. It is one thing that you don’t want to be informed and the other is that you cannot. I think those are the barriers. Also I have met people who know that they use a contaminant, and, for example, they don’t care. It is not important to them.* (age 36, translated from Spanish).

One mother suggested that women might feel like they might not be taken seriously. Other key concerns were “fear” and “privacy,” especially with regards to immigration status.

## Discussion

The purpose of this community-driven qualitative research was to engage urban Latina women living in environmental justice communities to identify and discuss health concerns related to perceived environmental hazards. Through this work, we ascertained detailed information about urban Latina women’s awareness of environmental hazards and whether and how this awareness influences behaviors, with specific attention to pregnancy and children.

In interviews with 36 Latina pregnant women and new mothers, we identified themes of *Defining the Environment*, *Environment & Health Risks*, and *Social & Political Responsibility*. The primary entry point to environmental health awareness among women interviewed was via tangible threats and lived experience – participants characterized adverse environmental exposures using everyday experiences with smell (e.g. bad odors), sight (e.g. visible smoke), sound (e.g. intense noise), and taste (e.g. disgusting water). Participants described environmental health as a collective concern and were frustrated by powerlessness to address potentially harmful environmental exposures.

There is limited research evaluating how pregnant women and mothers perceive environmental health risks, and few research groups have conducted qualitative research in this area [8, 26, 32–35]. Researchers in Ontario, Canada conducted interviews with fourteen new mothers [8] and fifteen mothers of young children [36]. Most existing research focuses on chemicals in consumer products, such as product use during pregnancy and breastfeeding among 22 mothers in Toronto, Canada [33], awareness of endocrine disrupting chemicals among pregnant women in Poitiers, France [37], perceptions of exposure to brominated flame retardants among pregnant women in Ontario, Canada [38], and concern about chemicals in consumer products among new mothers in Ohio, U.S. [39]. Researchers conducted focus groups with 103 Black and Latina women in the New York City neighborhoods of the South Bronx, Harlem, and Washington Heights [34] as part of the Healthy Home, Healthy Child campaign.

There are, however, some consistent themes among the Latina women in Los Angeles when compared with this literature. In both Canadian studies, as with ours, mothers describe becoming more aware of environmental health risks after having children and taking actions to protect their children from environmental hazards [8, 32, 33]. Women’s awareness of specific chemicals and hazards addressed by different studies is mixed, but across the board, mothers report using safer household products, eating organic foods, or controlling pests in their homes [8, 32, 33, 35, 40].

In prior qualitative studies, environmental quality was perceived as related to control and cleanliness, both indoors and outside. Mothers in Ontario expressed broad concerns about keeping their indoor environments “clean,” and felt that keeping their home clean was linked to a sense of control over their indoor environmental health [8]. Similarly, in New York City, women felt they had some aspect of control over their home environments and described efforts at keeping their homes clean and free of pests [34]. As with mothers in our study, women in New York City were also very aware of and concerned about the cleanliness of their outdoor environments, especially the presence of trash in their neighborhoods, and they expressed frustration with their lack of control over broader neighborhood environmental hazards like trash, air pollution, and building hazards [34]. While some Ontario mothers were less concerned about environmental health threats when they felt they had control over their environment, others accepted the risks in their outdoor environments *because* they had no control. Further, many of these mothers “demonstrated an optimistic bias,” describing how children in *other* cities or environments were at higher risk than their own [8].

Mothers’ environmental concerns, and their reported agency to address these concerns, are linked to socioeconomic and sociodemographic factors such as racism, immigration status, and wealth. In other studies, highly-educated, non-minority mothers with higher household incomes were more likely both to be concerned about the effects of chemicals in consumer products on their children’s health [39] and to report taking actions to protect their baby from environmental risks [32]. While these women described barriers to taking protective actions, including financial costs and lack of control [8, 32], mothers with higher income generally felt they had some control over their children’s exposure to chemicals in consumer products, especially around cleaning products and organic foods [33]. Mothers in Toronto described “complex precautionary consumption routines” to provide their children with non-toxic and chemical-free products, though only one “admitted to being overwhelmed” by the amount of information and decisions to make [33]. The predominantly low-income, urban Latina women in our study expressed similar feelings of concern about environmental health but had minimal agency to control their children’s environments. As with the participants we interviewed, mothers in Canada expressed concerns with the information they received about environmental health risks, with mistrust of some sources and frustration by the lack of specific actions they could take [8, 32].

Our study highlights the tension that many urban Latina mothers feel between wanting to protect their children and feeling powerless to change their environment because of power imbalances and infrastructure beyond their control. Although individual actions to reduce potentially harmful environmental exposures can be effective [41–45], results of individual interventions are inconsistent [46–48]. In one study, parental concern about environmental chemical exposures was associated with lower levels of some phthalates and BPA in their children, but higher levels of triclosan [39]. Moreover, in an effort to reduce their chemical exposures, consumers may encounter products with “regrettable substitutions” for known harmful chemicals – structurally and functionally similar chemicals with potentially similar adverse health impacts [49]. The media largely frames responsibility for pediatric environmental health risks as an individual responsibility [50]. However, the women we spoke to frequently expressed that individual actions were not within their reach. Further, while information about environmental exposures may encourage many women to take protective action, risk messages may also be a significant source of concern and stress, particularly if opportunities or resources required to take protective action are not readily available [51, 52].

Environmental health is highly context specific, and perceptions among different populations in the same place, or similar populations in a different place, may vary widely. As Scammell (2010) summarized, “context-specific social, cultural, and economic circumstances shape perceptions of environment and health, and the relationship between the two (i.e., environmental health)” [26]. The mothers in our current study identified as Latinas, immigrants, Spanish-speaking, women, primary caregivers, birth parents, and renters – multiply marginalized and intersecting identities [53–55]. An individual’s perception of risk informs how they respond to that risk [56]. Understanding how urban Latina pregnant women and mothers perceive their environment is important because they are disproportionately burdened by environmental health risks [5, 16, 17] due to historic and ongoing environmental racism and racial capitalism [57], a pattern that exists nationally [58]. Women in these communities face additional cultural stressors that, combined with environmental health risks, impact their health [59].

The results of this qualitative research can inform future quantitative studies [60]. There is limited documentation of urban Latina women’s perceptions of environmental health and how their prioritization of concerns align with outside individuals or organizations who aim to address environmental or health disparities. However, there is extensive documentation that the ways in which the public evaluates and perceives environmental health risk can be more complex and result in different conclusions than those of experts or “risk assessors” [60, 61]. Our results suggest that these mothers identified environmental health risks that may not be currently prioritized by researchers, funding agencies, or health professionals. For example, odors were a consistent concern identified throughout the course of the study, and perceptions of air pollution are frequently associated with measurable air pollution in environmental health research [62]. Self-reported odor or “annoyance” can reflect measurable levels of air pollutants [63–65]. However, there is limited monitoring or enforceable regulatory standards for chemicals that are not designated as criteria air pollutants [66], such as odor-causing chemicals like volatile organic compounds or hydrogen sulfide. Increased monitoring of these chemicals and prioritizing understanding how they may impact health and wellness at current levels could benefit communities.

Although this research was limited by a small convenience sample of participants, it is among the first to qualitatively evaluate urban Latina mothers’ perceptions of environmental health. The environmental health concerns raised by women in this study – odors, smog, trash, safety – should be incorporated in calls for regulatory action and enforcement at a policy level to reduce harmful chemical exposures [67, 68]. They are also important topics for future community based participatory research, which can further bridge local and scientific environmental health perceptions and expertise [69]. Community-engaged and community-driven models are shown to be more effective to advance public health than top-down strategies [70, 71] and more likely to be sustained when grounded in local systems and culture [72, 73]. The core theory of community organizing attests that social change happens when local residents develop and exert meaningful control over the social, economic, and political conditions in their neighborhoods. It is empowered, educated, and organized communities rather than “clients” or “subjects” that have consistently transformed communities [74]. These interviews increased our understanding of environmental risk perception and will inform future work on environmental health literacy.

## Conclusions

Latina mothers in Los Angeles are disproportionately burdened by exposure to environmental toxics, the effects of which may be amplified by other negative socioeconomic and social factors. Our interviews highlight the physical, tangible aspects of the environment that women perceive as contributing to or harming their environmental health, both in their communities and in their homes. Despite feelings of powerlessness to control their environments, mothers were vocal in their desires to learn more and to improve the environmental health in their communities. Policies and regulations that reduce environmental hazards among pregnant women and young children can be more effective than individual behavior changes to improve public health [75]. Multi-pronged, structural changes, as well as enforcement and expansion of existing environmental regulations, are needed to address the concerns of this vulnerable population. Community-engaged and community-driven research and educational tools can empower both community members and researchers to contribute to work that ultimately reduces and eliminates environmental health risks and disparities.

## Supplementary Information

**Additional file 1: **Interview Guide.

**Additional file 2: **Spanish quotes & translations.

## Data Availability

Deidentified data used during the current study are available from the corresponding author on reasonable request.
