# Supplementary material for: Perceptions and experiences of environmental health and risks among Latina mothers in urban Los Angeles, California, USA
Source: Environ Health. 2023 Jan 14;22:8. doi: 10.1186/s12940-023-00963-2 (PMC9840262; doi:10.1186/s12940-023-00963-2)
Supplement: Supplementary file 1 — Additional file 1: Interview Guide. [file 12940_2023_963_MOESM1_ESM.docx]

Appendix I. Interview Guide.

A few basic questions to begin:

- What is your age?
- Are your currently pregnant?
- How many children do you have? Please provide age and gender.
- What is your address? How many people live in your home with you? How many years have your lived at that address?
- Which state or country were you born in?

What I’d like to ask you to do is just talk to me about the environment and how it may relate to health in your community and house. When you hear the phrase environmental health – what do you think of?

*Basic Prompts:*

- *Anything else*
- *Can you tell me more*
- *Anything else – don’t worry about whether it’s right, just tell me what comes to mind*
- *Can you explain why*

Health

- What health concerns did you have during pregnancy?
- What health concerns did you have with your newborn/young child?
- How early in your pregnancy did you seek prenatal care? Did you stay at the same clinic for your entire pregnancy?

Environmental Health issues

- What sources of pollution are in your community? Which concern you the most?
- What sources of toxins are in your house? Which concern you the most?
- What kind of hazards, if any, are in your workplace?
- How can these environmental hazards impact the health of families, pregnant women and babies?
- Did you have any specific worries during pregnancy?
- Are you more or less concerned about the environment now that you are pregnant/have young children?
- Did you change your behavior or products you used during pregnancy? How about the products you use or don’t use with your young child?
- Among people living in your household, who is most concerned about the environment?

Research and Sources of information

- Where do you get your information? What information would be useful?
- How does scientific information influence you?
- Did you change your behavior based on this information or beliefs?
- Is environmental pollution an important source of risk in your community?
  - How do you think one can decrease exposure to chemicals?
  - Do you have concerns about any products your use in your home (like for cleaning or pest control)?
- What are interests in participating in research study? What are barriers?
