# Supplementary material for: Perceptions and experiences of environmental health and risks among Latina mothers in urban Los Angeles, California, USA
Source: Environ Health. 2023 Jan 14;22:8. doi: 10.1186/s12940-023-00963-2 (PMC9840262; doi:10.1186/s12940-023-00963-2)
Supplement: Supplementary file 2 — Additional file 2: Spanish quotes & translations. [file 12940_2023_963_MOESM2_ESM.docx]

Appendix II. Spanish quotes & translations

| Original quote | Translation for manuscript |
| --- | --- |
| Pues, por ejemplo que la comunidad sea limpia. Que sea segura para las familias y los niños. | *Well, for example that the community is clean. That it be safe for families and the kids.* |
| Lo que a veces yo pienso es por los cigarrillos, y por las drogas y los pandilleros, las balas. A veces por el agua, a veces llega sucia | *What I sometimes think about is cigarettes, and drugs, and gangs, and bullets. Sometimes about the water, sometimes it arrives to us dirty* |
| Cuando uno sale en la calle siempre hay personas que ve fumando en las paradas de los autobuses, en casi todo lugar donde uno sale siempre ve varias personas alli fumando. Y aunque uno quiere evitar eso siempre se encuentra en el lugar donde hay personas que fuman. Creo que ese es el problema | *When you go out on the street you always see people smoking at the bus stops, and* *everywhere you go outside you always see people smoking. And even though one may want to avoid it, one always finds people smoking. I think this is the problem* |
| La pintura, pues se esta cayendo pedazos de pintura y el manager no ase caso de pintar. Y así veo que el niño a veces jala la pintura...De salud si [I am worried], porque pues el polvo…[L]os niños a veces se les cae algo y se lo meten a la boca sin que uno se de cuenta…Mas peligro, entonces esa es la preocupación | *The paint, well, pieces are falling off and the manager does not want to repaint. And I see my child sometimes peel the paint...Regarding my health, yes [I am worried], because of the dust...sometimes something falls and the children put it in their mouths without us noticing...This is dangerous, and that is why there is worry.* |
| Las pesticidas? Lo que mata los insectos? Eso produce daño al cerebro… Por eso mata rápido a los animales y peligroso para los niños | *The pesticides – the ones that kill insects? It causes harm to the brain... that is why it kills animals fast and is dangerous for children.* |
| El agua también nos perjudica también. Si no está limpia, como cuando nos bañamos o tomamos si no está limpio nos perjudica nuestro organismo como Vómito, diarrea, calentura | *The water also harms us. If it [the water] is not clean, like when we shower or drink it, it can harm our body system like causing vomiting, diarrhea, and fever.* |
| Si hay mucho smog, y todo eso, pues que no es saludable | *If there is a lot of smog, and all that, well it is not healthy.* |
| Yo pienso que como todo ese humo todo eso le hace daño a uno. De repente a la salud porque de repente provoca mucha asma y todo eso. Muchas alergias | *I think that all that smoke can harm a person. Then suddenly it affects one’s health because it triggers asthma and a lot of allergies.* |
| porque hay mucho virus ahi de por lo que son los bichitos, mosquitas que se ponen ahi y todo eso…Pues igual trayendo pues alergias | *[B]ecause there is a lot of viruses from bugs, mosquitos that are around... and then they bring allergies* |
| [E]l embarazo es muy delicado. Tiene uno que tener mucho cuidado cuando esta uno embarazada para proteger al bebe y a uno | *[P]regnancy is very delicate. One has to be very careful when pregnant to protect the baby and oneself.* |
| Les afecta en muchos aspectos. Por que hay mujeres que al oler los químicos no pueden respirar. Y al no respirar uno afecta el bebe…Por que al momento que uno esta respirando muy fuerte el bebe también se esta agitando adentro | *It affects them in many aspects. For example, there are women who can’t breathe when they smell chemicals. And by not breathing, it affects the baby. Because the moment that you are breathing too hard the baby will be agitated inside.* |
| deformaciones o malformaciones | *Deformities or malformations* |
| [*Como piensa usted que estos peligros ambientales influyan en las salud de familias, mujeres embarazadas, y los bebes?]*  “Pienso que puede afectar mucho a los bebes, puede venir con una malformación o puede evitar también su crecimiento…puede afectarle mucho.” A 36 year old mother (102) | [How do you think these environmental hazards influence the health of families, pregnant women, and babies?] *I think that [environmental hazards] can affect babies very much. They can be born with a malformation or it can hinder their growth...it can affect them very much*. |
| No pues no. Todo normal. No senti que me afecto en eso | *Well no. Everything is normal. I didn’t feel it affect me*. |
| El humo de segunda mano afecta mas a los que recibe a el que lo esta haciendo. La gente lo sabe pero no respetan eso. No respetan de que están haciendo daño a otras personas eso es muy dañino y lo otro son los contaminantes de los carros pero eso no se puede evitar esta cuidad es muy contaminada por los carros | *Secondhand smoke affects those who do not smoke more than those who smoke. People know that but do not respect it. They do not respect that they are causing harm to other people. The other [source of pollution] is contaminants from cars but you can’t avoid that. This city is very contaminated by cars.* |
| Ellos trataron de entrar a fumigar mi apartamento, pero yo no les permiti porque la doctora me dijo que eso era malo para mis bebes y para mi. Entonces hasta me demandaron. Me querian correr porque no dejaba entrar. Pero gracias a Dios con la hoja que me dio el doctor me protegio. Y su demanda no siguio | *They tried to enter my apartment to fumigate it, but I didn’t let them because my doctor told me that it is bad for my kids and* *I. And they even sued me. They wanted to kick me out because I didn’t let them inside. But thank God, I was protected by the note my doctor gave me. And the lawsuit didn’t continue* |
| [A]qui los dueños de apartmetntos son…[laughing]…nos les importan…de lo que les importa es que les estamos dando la renta | *[H]ere the managers of the apartments are...[laughing]...they don’t care...they only care about receiving rent* |
| [P]or mucha promoción que le estén haciendo, no abunda el dinero. Porque tan solo en unas dos canastita de fresitas, o tres, ya son cinco dólares. En un ramo de espinacas ya son dos dólares. Entonces la promoción no sirve de nada porque de todos modos se esta yendo el dinero | *For all the deals they are having, the money does not go far enough. Because only two baskets of strawberries, or three, it is already five dollars. A spinach bunch is already two dollars. So, then the deal is useless because the money does not last.* |
| Yo estaba trabajando en la costura y en ese trabajo las telas traen mucho polvo entonces cuando uno cose la ropa entonces le sale much polvo y a veces le entra a uno por la nariz, por los oidos y los ojos. Y a veces a uno le cuesta respirar. …No, a uno no le dan nada [protective gear] y uno gana bien poquito. Porque a veces uno gana cinco centavos, diez centavos, quince centavos a la pieza y uno no hace muchas pieza pero uno se llena de polvo | *I was working in garment factory and in that job the fabrics make a lot of dust so when you sew clothes it creates dust and sometimes the dust enters you through your nose, ears and eyes. And sometimes you find it hard to breathe. …No, they don't give you anything [protective gear] and one earns very little. Because sometimes you earn five cents, ten cents, fifteen cents per piece and you do not make many pieces but you get full of dust* |
| Antes yo compraba mas cloro que todo. Ahora solo uso el jabón y bicarbonate | *Before I would buy more bleach for everything. Now I only use soap and baking soda.* |
| pero son bien altos los precios y una familia de bajos ingresos no puedo comprar eso productos | *[B]ut the prices are very high and a low-income family cannot buy those products.* |
| Por ejemplo, yo soy de un pueblo, ahí cuando cortan un árbol siembran 10 árboles. Entonces como el ecosistema el paisaje lo van aumentando más. Aquí cuando ya no hay árboles ya no hay nada, no hacen por sembrar algo más | *For example, I am from a town, where when they cut down a tree they plant 10 more trees. That way the ecosystem, the scenery keeps growing. Here when there are no trees there is nothing, they don’t do anything to change that.* |
| Me gustaría que quitaran la cosa de petróleo para que no viera mas contaminantes…Y el exhuesion de los automóviles… Todo es contamina. | *I would like them to remove the petroleum wells so that that there are no more contaminants… And the exhaust from the cars… All of this is polluting.* |
| “[h]ay lugares también donde se desperdicie mucha agua. Dejan regando las plantas asi toda la noche… que no hay mucha agua y que se esta acabando el agua que se puede consumer. | *[T]here are places where a lot of water is wasted. They leave the water running all night… there is not much water and the water that can be consumed is running out* |
| Pienso que tengo que tratar de no tirar cosas por bien de los niños por que a nosotros ya nos toco a vivir pero a ellos toda vía les toca a vivir con un ambiente limpio y sano también | *I think I have to try not to throw things [away] for the good of the children because we already got to live but they still need a chance to live in a clean and healthy environment too.* |
| “[p]orque me imagino que con las repuestas que de uno van a basarse para tratar de ayudar a la comunidad con mas programas, con mas cosas | *[B]ecause I imagine that the answers that one provides will be used to help the community with more programs.* |
| Por ejemplo, para no ir al doctor. Yo pienso y siento de que tiene mucho quimicos la medicina. Entonces digo no, pues. Le hago un te que es natural | *For example, to not go to the doctor. I think and I feel that medicine has a lot of chemicals. So I say no. Instead, I make a natural tea* |
| Pero las otras madres, en cambio, están trabajando. Se enfocan solo en el trabajo. Llegar a su casa, ir al trabajo, y otra vez a la casa. Entonces eso es uno de los factores. Uno es que no se quieren informar y otra es que no pueden. Pienso yo que esas son las barreras. Y las otras son es que he conocido ha gente que saben que usan un contaminante y por ejemplo les vale. No les importa. | *But the other mothers, in contrast, are working. They focus only on work, then go home, and go to work, and home again. So that's one of the factors. It is one thing that you don't want to be informed and the other is that you cannot. I think those are the barriers. Also I have met people who know that they use a contaminant, and, for example, they don’t care. It is not important to them* |
